# Synergistic effect of arginine and *Lactobacillus plantarum* against potassium dichromate induced-acute liver and kidney injury in rats: Role of iNOS and TLR4/NF-κB signaling pathways

**DOI:** 10.22038/IJBMS.2023.68855.15108

**Published:** 2023

**Authors:** Ahmed A. Sedik, Azza Hassan, Abeer Salama

**Affiliations:** 1 Pharmacology Department, Medical Research and Clinical Studies Institute, National Research Centre, 12622, Egypt; 2 Pathology Department, Faculty of Veterinary Medicine, Cairo University

**Keywords:** Arginine, Caspase-3, Inos, Lactobacillus plantarum, potassium dichromate, TLR 4/NF-κB signaling-pathway

## Abstract

**Objective(s):**

Our study was conducted to evaluate the synergistic effect of arginine (ARG) and *Lactobacillus plantarum* against potassium dichromate (K2Cr2O7) induced-acute hepatic and kidney injury.

**Materials and Methods:**

Fifty male Wistar rats were divided into five groups. The control group received distilled water. The potassium dichromate group (PDC) received a single dose of PDC (20 mg/kg; SC). The arginine group (ARG) and *Lactobacillus plantarum* group received either daily doses of ARG (100 mg/kg, PO) or *L. plantarum* (10^9^ CFU/ml, PO) for 14 days. The combination group (ARG+*L. plantarum*) received daily doses of ARG (100 mg/kg) with *L. plantarum* (10^9^ CFU/ml), orally for 14 days, before induction of acute liver and kidney injury. Forty eight hours after the last dose of PDC, serum biochemical indices, oxidative stress biomarkers, pro-inflammatory cytokines, histopathological and immunohistochemical analysis were evaluated.

**Results:**

Combining ARG with *L. plantarum* restored the levels of serum hepatic & kidney enzymes, hepatic & renal oxidative stress biomarkers, and TLR 4/ NF-κB signaling pathway. Furthermore, they succeeded in decreasing the expression of iNOS and ameliorate the hepatic and renal markers of apoptosis: Caspase-3, Bax, and Bcl2.

**Conclusion:**

This study depicts that combining ARG with *L. plantarum* exerted a new bacteriotherapy against hepatic and renal injury caused by PDC.

## Introduction

Potassium dichromate (PDC) is heavy metal chromium (VI) and a strong oxidizing toxic agent. It is used in paints, the manufacture of magnetic tape, the tanning of leather, and as a catalyst for hydrocarbon production. The exposure to chromium (VI) is highly toxic and widely distributed in the environment inducing carcinogenic and mutagenic effects on human beings and animals (1). In addition, chromium (VI) work-related exposure among millions of workers leads to serious injuries in the liver and kidney (2) and other vital organs such as the lung (3) and brain (4). 

PDC mediates the inflammatory process by elevating pro-inflammatory cytokine as tumor necrosis factor-alpha (TNF-α) (5). Overproduction of TNF-α and IL-1β promotes the inflammatory immune response to tissue damage (6), which in turn activates the immune system and inflammatory response, which are responsible for the development of both acute liver and renal disease (7, 8). Some immune cells and renal cells induce inflammatory response through the expression of proinflammatory cytokines; TNF-α and interleukin-6 (IL-6) which disrupt renal cells’ cellular structure and biological function causing kidney injury and cell death (9). On the other hand up-regulation of the release of TNF-α and interleukin-1 beta (IL-1β) by Kupffer cells and stimulated hepatic stellate cells (HSCs) exhibit liver disorder (10).

Toll-like receptor-4 (TLR-4) was suggested as an important element in the immune system and it is considered a causative factor for inflammatory cytokine release. TLR-4 responds to the toxin-stimulating host defense (11). TLRs stimulate innate immunity and are involved in renal injury pathogenesis, vascular remodeling, and endothelial dysfunction (12). Activated TLR-4 is a tubular damage inducer via activating nuclear factor-kappa B (NF-κB) and stimulates renal damage and fibrosis (13). PDC induced renal injury via elevating gene expression of TLR-4 protein, which is involved in innate and adaptive immune response and constantly increased TNF-α (14).

Inducible nitric oxide synthase (iNOS) is a powerful free radical that produces nitric oxide (NO) over time and its active metabolite, peroxynitrite (15). The activation of iNOS has been associated with several signaling pathways, including the NF-κB transcription factor. NF-κB is a cytoplasmic transcription factor that is dormant and is involved in the squeal of various inflammatory responses (16). It is activated by a variety of external stimuli, including reactive oxygen species (ROS), and binding to the corresponding DNA sequence of the target genes causes cell and tissue damage (17). TLRs are pattern recognition receptors that respond to endogenous danger signals and are involved in the development of acute liver and kidney injury (18). TLR4 activates the NF-κB signaling pathway, which results in the generation of cytokines and the expression of co-stimulatory molecules (19). Therapeutic approaches that modulate the above signals could be potential prospects for treating a variety of pathological conditions.

Arginine (ARG) amino acid stimulates systemic immune function. It is obtained from the diet although it can be synthesized in mammals (20). A decrease in arginase activity is an inducer of non-alcoholic fatty liver disease development (21). ARG administration overcomes paracetamol-induced toxicity in the liver (22). Its supplementation, also, has good protective effects against renal failure through modulation of the nitric oxide pathway (23). It has a protective role against renal damage induced by lead and cadmium as it is capable of inhibiting lipid peroxidation processes and causing an elevation in glutathione enzyme activity (24). 

Probiotics have been reported to enhance immunity by activating liver and peritoneal macrophages (25). *L.* *plantarum* is among the most important probiotics and exhibited an overall strong regulatory effect on liver damage induced by ethanol in mice (26). *L.* *plantarum *has therapeutic potential against acetaminophen-induced kidney damage in experimental animals. It has a potent antioxidant effect in suppressing endothelial apoptosis induced by oxidants (27). The use of probiotics, alone or in combination with other therapies has potential benefits due to the mucosal action of probiotics and the systemic effect of ARG (28); so, the present study was designed to evaluate the effect of ARG alone or in conjunction with *L.* *plantarum* to combat PDC-induced acute liver and kidney injury in rats.

## Materials and Methods

***Experimental animals***

Fifty male Wistar rats weighing about (180-200 g) were obtained from the Animal National Research Centre (Egypt). Rats were kept in a well-ventilated room at ambient laboratory conditions (22-28 ^°^C temperature, 45-55% relative humidity) with a photoperiod of a 12-hr light/12-hr dark cycle. Food and water were available *ad libitum*. The animal experiments were performed using the recommendations in the Guide for the Care and Use of Laboratory Animals of the National Institutes of Health (NIH No. 85.23: revised 1985) and approved by the Medical Research Ethics Committee (MREC) of the National Research Centre (Egypt) (approval number 5413042022).

***Bacterial strain and chemicals***

*L.* *plantarum* 4496 was obtained from Northern Regional Research Laboratory (NRRL, USA). Arginine (ARG), CAS No: 74-79-3 (purity, 99%), and potassium dichromate (K2Cr2O7), CAS No: 7778-50-9 (purity, 99%) were purchased from Sigma Aldrich (USA). All other reagents and kits were obtained from Biodiagnostic and Randox companies.

***Growth conditions***

*L.* *plantarum* 4496 strain was activated in De Mann Rogosa Sharpe (MRS) broth for 48 hr under anaerobic conditions at 37 ^°^C, then was inoculated in sterilized skimmed milk with a concentration of 3% w/v and incubated at 37 ^°^C for 24 hr. The daily oral administration of the *L.* *plantarum* was (1×10^9^ CFU/ml).

***Experimental protocol***

**Figure 1:**
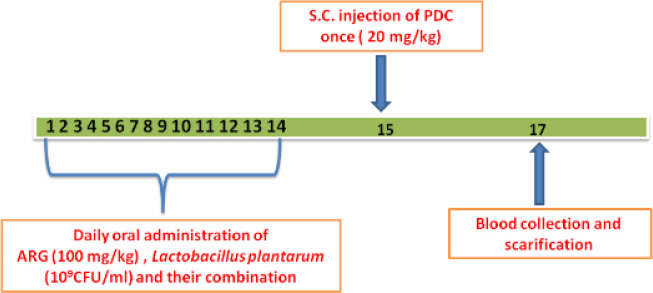
Schematic diagram illustrating the experimental design of acute hepatic and kidney injury induced by PDC in rats

Rats were randomly divided into five equal groups (10 rats /group) as follows (Figure 1):

Control group: Rats were orally administrated distilled water daily for 14 days.

Potassium dichromate group (PDC) group: Rats were subcutaneously injected with PDC once at a dose of 20 mg/kg and served as a control-positive group (29).

Arginine group (ARG): Rats were orally pre-treated with ARG (100 mg/kg) daily for 14 days before induction of acute liver and kidney injury with PDC (30).

*Lactobacillus plantarum* group: Rats were orally pre-treated with *L.* *plantarum* (10^9 ^CFU/ml) daily for 14 days before induction of acute liver and kidney injury with PDC (31).

Combination group (ARG+*Lactobacillus plantarum): *Rats were orally pre-treated with ARG (100 mg/kg) and *L.* *plantarum* (10^9 ^CFU/ml) daily for 14 days before induction of acute liver and kidney injury with PDC.

***Serum and tissue sample collection and preparation***

By the end of the experiment, rats were anesthetized with pentobarbital sodium, blood samples were collected from the retro-orbital venous plexus (under inhalation anesthesia of isoflurane) and centrifuged at 1500 rpm for 10 min for serum separation and then kept at -20 ^°^C for further serum biochemical analyses (32, 33). After sacrificing of rats by cervical dislocation, the liver and kidneys from each rat were excised immediately and divided into two parts: part one was kept at -80 ^°^C for further tissue biochemical analysis, while part two was fixed in 10% neutral formalin for histopathology and immunohistochemistry examinations. 

The preserved liver and kidney from each rat were washed in normal saline solution and blotted over filter paper and then perfused in a cold phosphate-buffered saline (PBS; pH 7.4) containing 0.16 mg/ml heparin to remove blood clots. Then 1 g of tissues was homogenized in 9 volumes of cold PBS (pH 7.4) using an automatic tissue homogenizer (Heidolph, Germany). The tissue homogenate was centrifuged at -80 ^°^C and 4000 rpm for 15 min, and the supernatant was collected and kept at -80 ^°^C for the measuring of reduced glutathione (GSH), malondialdehyde (MDA), and catalase levels.

***Assessment of serum hepatic and renal function indices***

Serum levels of aspartate transaminase (AST), alanine transaminase (ALT), alkaline phosphatase (ALP), urea, creatinine, and uric acid were evaluated using colorimetric kits purchased from Biodiagnostic Company (Dokki, Giza, Egypt). AST (CAT.NO. AS1061 (45), ALT (CAT. NO. AS 1031(45), ALP (CAT.NO. AP 10 20) Urea (CAT.NO.UR2110), creatinine (CAT.NO.CR1250), and uric acid (CAT.NO. UA 21 20) were performed according to the manufacturer’s instructions (34-38).

***Assessment of hepatic and renal oxidative stress biomarkers***

Oxidative stress biomarkers, including reduced GSH, MDA, and catalase levels were assessed in hepatic and kidney tissues homogenate using commercial kits of GSH (CAT.NO. GR 2511), MDA (CAT.NO.MD 2529), and catalase (CAT.NO. 2533) that were supplied by the Biodiagnostic Company (39-41).

***Assessment of hepatic and renal levels of NF-κB, TLR4, and IL-1β***

Hepatic and renal levels of NF-κB, TLR4, and IL-1β were estimated using a rat NF-κB ELISA kit (Sunlong Biotech Co., Catalog no. SL0537Ra, CHINA), rat TLR4 ELISA Kit (Sunlong Biotech Co., Catalog no. SL0699Ra, CHINA), and rat IL-1β ELISA Kit (Sunlong Biotech Co., Catalog no. SL0402Ra, CHINA), depending on the sandwich-ELISA method. The optical density (OD) for the concentration of NF-κB, TLR4, and IL-1β were measured spectrophotometrically at the wavelength of 450 nm (42-44). 

***Assessment of hepatic and renal levels of TNF-α and caspase-3***

Levels of TNF-α and caspase-3 were assessed in hepatic and kidney homogenate of rats using the rat TNF-α ELISA kit (Sunlong Biotech Co., Catalog no. SL0722Ra, CHINA) and Caspase 3 ELISA Kit (Sunlong Biotech Co., Catalog no. SL0152Ra, CHINA), depending on the sandwich-ELISA method. The optical density (OD) for the concentration of TNF-α and caspase 3 was measured spectrophotometrically at the wavelength of 450 nm (45, 46).

***Histopathological examination of hepatic and renal tissues***

Hepatic and kidney specimens were excused and fixed in 10% neutral buffered formalin to be further stained with hematoxylin and eosin stain (H&E)(4 µm thick sections). The specimens were photographed using a digital Leica photomicroscope (LEICA DMLB Germany) (47). 

***Immunohistochemical analysis of Bax, Bcl-2, and iNOS in hepatic and renal tissues***

The immune-staining method for localization of Bax (Bcl-2-associated X), B-cell lymphoma-2 (Bcl-2), and iNOS was performed (48). The formalin-fixed hepatic and renal sections were deparaffinized, rehydrated in different alcohol concentrations, incubated in 3% H_2_O_2_, and then incubated overnight at 4 ^°^C as primary antibodies with anti-Bax (diluted 1:100, Santa Cruz Biotechnology Santa Cruz, CA, USA), anti-Bcl-2 (diluted 1:200, Santa Cruz Biotechnology, Santa Cruz, CA, USA), and anti- iNOS (diluted 1:200, Abcam, Cambridge, MA, USA). After rinsing with phosphate-buffered saline, they were incubated with appropriate biotinylated secondary antibodies according to the Vecta stain Elite ABC Kit (Vector Laboratories, Burlingame, CA, USA) for 30 min at room temperature. The immune reaction was visualized using diaminobenzidine (DAB, Sigma Chemical Co, St. Louis, Missouri, USA). The samples were scored (% of positive cells/HPF) (49, 50).

***Statistical analysis***

The results are expressed as mean±SEM (8) rats and all measurable comparisons were made by using one-way analysis of variance (ANOVA) followed by Tukey’s multiple comparisons test. The data were analyzed with GraphPad Prism v. 8.0 (GraphPad Software, Inc., CA, USA). The difference was reported as significant when the *P*-value is ≤0.05. The interaction between ARG and *L.* *plantarum* was evaluated by measuring the combination index (CI), a quantitative pharmacological tool between two drugs using CompuSyn 1.01 software (ComboSyn, Inc., Paramus, NJ, USA) where, CI<1, CI=1, and CI>1 were considered to indicate synergistic, additive, and antagonistic effects, respectively (51). This method has been used in various previous studies (52, 53).

## Results

***Pre-treatment effect of combining ARG with Lactobacillus plantarum on serum biochemical indices in rats receiving PDC induced-acute hepatic and kidney injury***

Rats receiving a subcutaneous dose of PDC (20 mg/kg) showed an increase in serum AST, ALT, ALP, urea, creatinine, and uric acid levels of nearly 250%, 230%, 399%, 317%, 316%, and 170% of the normal value, respectively. Pre-treatment of rats receiving PDC with ARG (100 mg/kg) orally for 14 days showed a decrease in the serum levels of AST, ALT, ALP, urea, creatinine, and uric acid levels nearly 223%, 155%, 203%, 207%, 165%, and 145% of the normal value, respectively. Pre-Treated rats receiving PDC with *L.* *plantarum* (10^9 ^CFU/ml) orally for 14 days revealed decreased values of AST, ALT, ALP, urea, creatinine, and uric acid roughly 147%, 118%, 174 %, 182%, 146%, and 127% of the normal value, respectively. Pre-treatment of PDC rats with (ARG+*L. plantarum)* for 14 days could restore the aforementioned parameters to normal values (Table 1). Furthermore, combining ARG with *L. plantarum* revealed a synergistic effect on the levels of AST, ALT, ALP, urea, creatinine, and uric acid elucidated by the calculated CI values which are CI<1 (0.17, 0.63, 0.73, 0.51, 0.81, and 0.7, respectively).

***Pre-treatment effect of combining ARG with Lactobacillus plantarum on hepatic and renal oxidative stress biomarkers in rats receiving PDC induced-acute hepatic and kidney injury***

Injected rats with PDC (20 mg/kg; SC) showed an increase in hepatic and renal values of MDA of nearly 209% and 218% of the normal value, respectively, and with a decrease in the hepatic and renal activity of catalase reaching nearly 74% and 66% of the normal value, respectively in addition to a decrease in hepatic and renal GSH activity nearly 36% and 46% of the normal value, respectively. Pre-treatment of rats receiving PDC with ARG (100 mg/kg) orally for 14 days showed a decrease in hepatic and renal values of MDA reaching about 121% and 128% of the normal value, respectively, with an increase in hepatic and renal activity of catalase reaching nearly 96% and 101% of the normal value, respectively in addition to an increase in hepatic and renal GSH activity nearly 90% and 84% of the normal value, respectively. Pre-treated rats receiving PDC with *L. plantarum* (10^9 ^CFU/ml) for 14 days revealed a decrease in hepatic and renal values of MDA reaching about 138% and 164% of the normal value, respectively, with an increase in hepatic and renal activity of catalase reaching nearly 96% of the normal value, in addition to an increase in hepatic and renal GSH activity nearly 72% of the normal value. Combining ARG with *L. plantarum* for 14 days could restore the aforementioned parameters to normal values (Table 2). Moreover, combining ARG with *L. plantarum* revealed a synergistic effect on the levels of hepatic and renal GSH, MDA, and catalase evidenced from the calculated CI<1 as follows; 0.35, 0.7, 0.33, 0.10, 0.5, and 0.47, respectively.

***Pre-treatment effect of combining ARG with Lactobacillus plantarum on the levels of NF-*** ***κ*** ***B, Il-1β, and TLR-4 in the liver and kidney of PDC-induced acute hepatic and kidney injury in rats***

**Figure 2:**
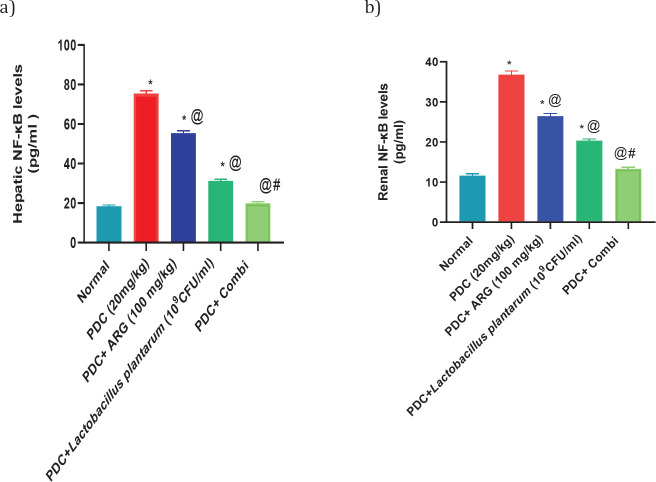
Pre-treatment effect of ARG in combination with *Lactobacillus plantarum* on the levels of NF-κB in the liver and kidney of rats receiving PDC

**Figure 3:**
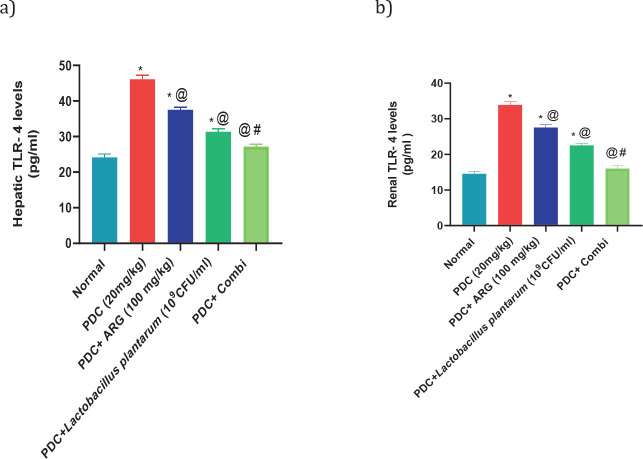
Pre-treatment effect of ARG in combination with *Lactobacillus plantarum* on the levels of TLR- 4 in the liver and kidney of rats receiving PDC

**Figure 4:**
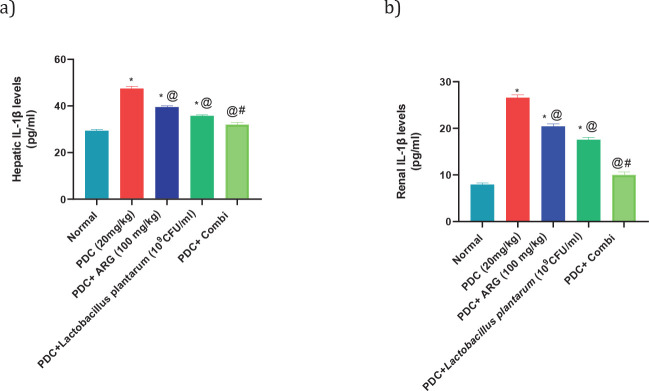
Pre-treatment effect of ARG in combination with *Lactobacillus plantarum* on the levels of il-1β in the liver and kidney of rats receiving PDC

Subcutaneous injection of PDC at a dose of 20 mg/kg showed an increase in hepatic levels of NF-κB, Il-1β, and TLR-4 nearly 4 folds, 162% and 190% of the normal value, respectively, and consequently in the renal levels of NF-κB, Il-1β, and TLR-4 nearly 316%, 335% and 233% of the normal value, respectively. Rats receiving pre-treated doses of ARG (100 mg/kg; PO) showed a decrease in hepatic levels of NF-κB, Il-1β, and TLR4 nearly 3 folds, 134% and 155% of the normal value, respectively, and consequently in the renal levels of NF-κB, Il-1β, and TLR-4 nearly 227%, 257%, and 189% of the normal value, respectively. Rats receiving pre-treated doses of *L. plantarum* showed a decrease in hepatic levels of NF-κB, Il-1β, and TLR-4 by nearly 170%, 121%, and 129% of the normal value, respectively, and consequently in the renal levels of NF-κB, Il-1β, and TLR4 nearly 174%, 221%, and 154% of the normal value, respectively. Combining ARG with *L. plantarum* in PDC rats for 14 days could restore the hepatic and renal values of NF-κB, Il-1β, and TLR4. Furthermore, combining ARG with *L. plantarum* revealed a synergistic effect on hepatic and renal levels of NF-κB, Il-1β, and TLR-4 elucidated by the calculated CI values which are CI<1 (0.55, 0.26, 0.39, 0.20, 0.37, and 0.27, respectively)(Figures 2, 3, and 4). 

***Pre-treatment effect of combining ARG with L. plantarum on the levels of TNF-α and caspase-3 in the liver and kidney of PDC-induced acute hepatic and kidney injury in rats***

**Figure 5:**
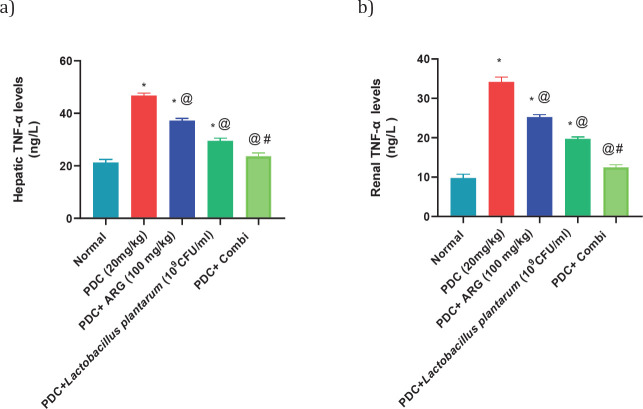
Pre-treatment effect of ARG in combination with *Lactobacillus plantarum* on the levels of TNF-α in the liver and kidney of rats receiving PDC

**Figure 6:**
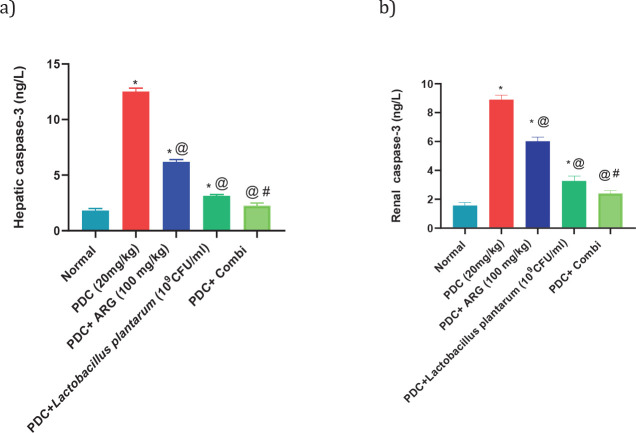
Pre-treatment effect of ARG in combination with *Lactobacillus plantarum* on the levels of Caspase-3 in the liver and kidney of rats receiving PDC

Injection of PDC to rats at a dose of 20 mg/kg showed an increase in hepatic levels of TNF-α and caspase-3 nearly 2 folds and 6 folds of the normal value, respectively, and consequently in the renal levels of TNF-α and caspase-3 nearly 3.5 folds of the normal value. Rats receiving pre-treated doses of ARG (100 mg/kg; PO) showed a decrease in hepatic levels of TNF-α and caspase-3 by nearly 175% and 176 folds of the normal value, respectively, and consequently in the renal levels of TNF-α and caspase-3 nearly 201% and 176% of the normal value, respectively. Rats receiving pre-treated doses of *L. plantarum* showed a decrease in hepatic levels of TNF-α and caspase-3 by nearly 138% and 176% of the normal value, respectively, and consequently in the renal levels of TNF-α and caspase-3 nearly 2 folds of the normal value, respectively. Combining ARG with *L. plantarum* in PDC rats for 14 days could restore the hepatic and renal values of TNF-α and caspase-3 (Figures 1 and 2). Furthermore, combining ARG with *L. plantarum* revealed a synergistic effect on hepatic and renal levels of TNF-α and caspase-3 elucidated by the calculated CI values which are CI<1 (0.44, 0.29, 0.39, and 0.29, respectively)(Figures 5 and 6). 

***Pre-treatment effect of combining ARG with L. plantarum on histopathology of liver and kidney in rats receiving PDC induced-acute hepatic and kidney injury***

**Figure 7:**
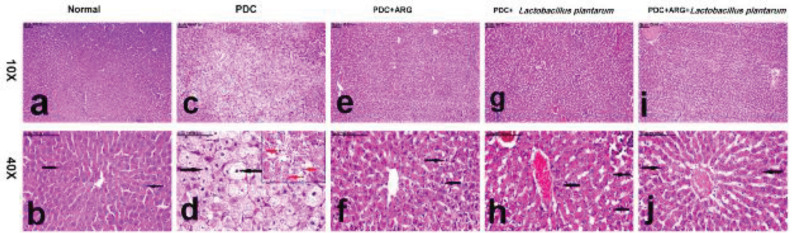
Pre-treatment effect of combining ARG with *Lactobacillus plantarum* on the histopathological changes in the liver of rats that received PDC

**Figure 8:**
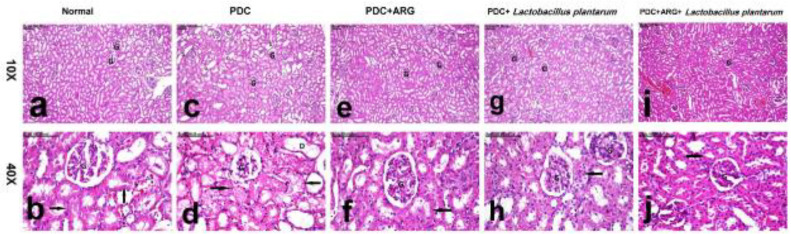
Pre-treatment effect of combining ARG with *Lactobacillus plantarum* on the histopathological changes in the kidney of rats that received PDC

The total pathologic score recorded in the liver and kidneys of all groups is illustrated in Table 3. Microscopic examination of the liver and kidney of the normal group showed normal hepatocytes, with round vesicular nuclei with no evidence of hepatocellular swelling. Similarly, the kidneys of normal rats showed normal tubules with normal epithelial lining and normal round vesicular nuclei. In contrast, the Liver of the PDC group revealed pronounced swelling of hepatocytes with cytoplasmic reticulation and nuclear pyknosis. The markedly ballooned hepatocytes were interspersed with abundant necrotic and apoptotic cells. Additionally, extensive renal damage was demonstrated in the PDC group, in the form of extensive necrosis of renal tubules with small deep bluish pyknotic nuclei. In addition to the presence of hyaline and epithelial casts in the lumen of some renal tubules. Markedly dilated renal tubules with epithelial spreading were a common histopathological finding in the PDC group. Very remarkable amelioration was recorded in the liver and kidneys of the PDC+ARG group, in which hepatocytes appeared less remarkably swollen with mild vacuolar degeneration of hepatocytes. Similarly, normal renal tubules were demonstrated in nearly all examined sections, with no evidence of acute cellular swelling, tubular necrosis, or tubular dilatation. In like manner, the liver of the PDC+*Lactobacillus plantarum* group showed mild vacuolation of hepatocellular cytoplasm, with no evidence of cellular ballooning. Only individual cell necrosis was demonstrated in the kidneys of this group and the remaining renal tubules appeared normal in addition to the presence of mitotic figure denoting regenerative activity. On the other hand, normal liver and kidneys were demonstrated in the combination group, with a marked decrease in total pathologic score (Table 3). The hepatocytes and renal tubules appeared normal with normal round vesicular nuclei (Figure 7 for the liver and Figure 8 for the kidney).

***Pre-treatment effect of combining ARG with Lactobacillus plantarum on levels of Bax and Bcl-2 and is in the kidneys of PDC-induced acute hepatic and kidney injury in rats***

The results of immunohistochemical analysis recorded in the liver and kidneys of normal and other treated groups are illustrated in Table 4.

***Bax expression***

**Figure 9:**
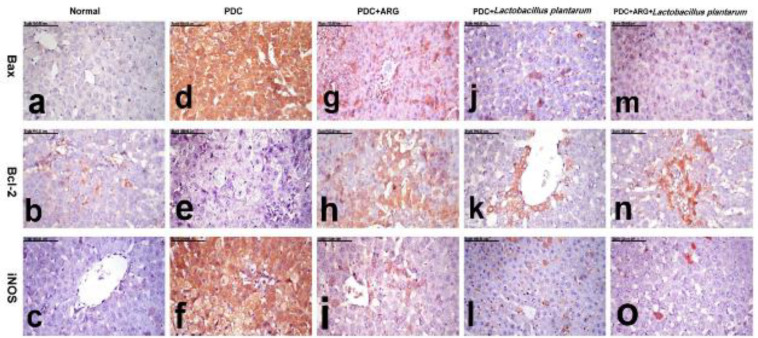
Pre-treatment effect of combining ARG with *Lactobacillus plantarum* on levels of Bax, Bcl-2, and iNOS in the liver of rats receiving PDC

**Figure 10:**
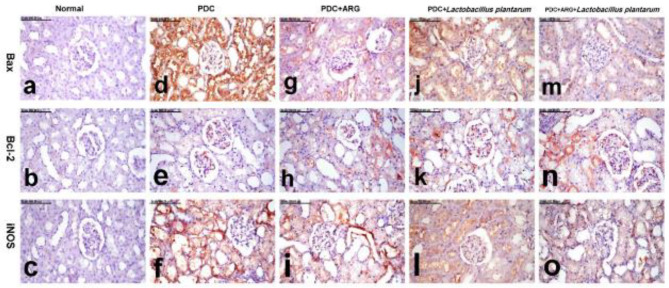
Pre-treatment effect combining ARG with *Lactobacillus plantarum* on levels of Bax, Bcl-2, and iNOS in the kidney of rats that received PDC

No Bax expression was demonstrated in the liver and kidneys of the normal group. In contrast, Bax expression was significantly increased in the liver and kidneys of the PDC group. Bax immune stained cells revealed strong brown cytoplasmic staining. A significant reduction of Bax expression, with a significant decrease of percentage of positively stained cells, was recorded in the livers and kidneys of the combination group. The pronounced decrease of Bax expression was recorded in the combination group (ARG+*Lactobacillus plantarum*) (Figure 9 for the liver and Figure 10 for the kidney).

***Bcl-2 expression***

Few Bcl-2 positively stained cells were demonstrated in the liver of the normal group, but no pronounced Bcl-2 expression was demonstrated in the kidneys of the normal group. A pronounced decrease of Bcl-2 expression was recorded in the liver and kidneys of the PDC group, in which sparse Bcl-2 weakly stained cells were demonstrated in the liver and renal glomeruli. On the contrary, a significant increase of percentage of Bcl-2 positively stained cells was recorded in the liver and kidneys of the PDC+ARG and PDC+*Lactobacillus plantarum* group. On the other hand, a significant increase of Bcl-2 expression with an increased percentage of positively-stained cells was recorded in the liver and kidneys of the combination group (ARG+*Lactobacillus plantarum*) (Figure 9 for the liver and Figure 10 for the kidney).

***iNOS expression***

The liver and kidneys of the normal group revealed no iNOS expression. But, the liver and kidneys of the PDC group revealed a significant increase in iNOS expression, with numerous intense iNOS immune-positive cells. A pronounced decrease of iNOS expression, with a significant decrease of percentage of iNOS-positively stained cells, was recorded in the liver and kidneys of the PDC+ARG group and PDC+*Lactobacillus plantarum* group, with a non-significant difference between them. An obvious reduction of iNOS expression, with an outstanding reduction of percentage of positively stained cells, was demonstrated in the liver and kidneys of the combination group, which revealed individual sparse iNOS immune-positive cells (Figure 9 for the liver and Figure 10 for the kidney). 

## Discussion

Heavy metal pollution, due to industrial activities, is increasing worldwide. Its occupational exposure happens in chrome-plating workers, welders, and chromium pigment factory workers, inducing acute liver (54), kidney (55), and brain injury (56). Therefore, the present study aimed to investigate the possible protective effects of ARG and /or *L. plantarum* on liver and kidney toxicity, inflammation, and apoptosis induced by PDC intoxication on the liver and kidneys in rats. This metal induced disturbance in hepatic enzyme biosynthesis and alteration in the hepatic membrane permeability that was confirmed in this study by a significant increase in ALT, AST, and ALP in serum, with a reduction in kidney function, which was reflected by the elevation in serum levels of urea, creatinine, and uric acid. Chromium in previous work, increased serum ALT activity, proteinuria, creatinine, and urea nitrogen causing hepatotoxicity, and nephrotoxicity (57). These elevations confirm the cellular leakage existence of these parameters and may indicate cell membrane injury (58). 

ARG or *L.* *plantarum*, in the current study, had protective effects on liver and kidney injury induced by PDC in rats that are evidenced by a reduction in liver and kidney functions as compared with PDC rats, in addition, the combination of ARG with *L.* *plantarum* has beneficial effects on liver and kidney injury as it returned serum biochemical indices to their normal levels. These findings suggest that the combination of ARG preserved the hepatocyte integrity more than that produced by each treatment. Previously, ARG decreased ALT and AST inhibiting liver ischemia-reperfusion injury in a rat model (59). Moreover, *L.* *plantarum* has protective effects on liver and kidney damage by endosulfan in pregnant rats (60). *L.* *plantarum*, also, has therapeutic potential against acetaminophen-induced uremia in rats through decreasing kidney function, DNA damage, and glomerular necrosis (61). Furthermore, a single SC injection of PDC released oxidative and nitrosative stress markers, as it is a strong oxidant (62). Our results exhibited that PDC caused oxidant and antioxidant imbalance that was supported by an elevation in renal and hepatic levels of MDA and reductions of GSH and catalase while the combination of ARG with *L.* *plantarum* restored these biochemical indices to their normal levels. Previously ARG supplements reduced MDA levels, and elevated GSH and catalase in renal and liver injury in rats with myocardial infarction (63). Also, *L. plantarum* has an antioxidant effect against oxidized oil-induced hepatic injury (64). 

TLR4, a transmembrane protein, is expressed in monocytes and macrophages, including Kupffer cells. Where, it mediates Kupffer cell activation by lipopolysaccharide (LPS)-induced liver injury, and is involved in the mechanism of alcohol-provoked hepatic disorder (65). Moreover, TLR4 is an important key in controlling inflammatory response and activates the innate immune that is involved in the pathogenesis of kidney injury induced by PDC, initiating the release of cytokine TNF-α, inducing renal inflammatory response (66). TNF-α plays a crucial role in mediating the interplay between inflammatory, oxidative stress, and apoptotic pathways (67). Our study revealed that PDC induces a massive inflammatory response in liver and renal tissues which was observed by the elevation of TNF-α (68). Combining ARG with *L. plantarum* significantly inhibited TNF-α, which can be assumed as a chief mechanism for its protective role in PDC-induced acute hepatic and kidney injury. This finding is in line with the previous study that exhibited their protective effects against endotoxin-induced liver injury (69).

At the same time, NF-kB activates the production of inflammatory mediators such as TNF-α, IL-1β, iNOS, and COX inducing the regression of the inflammation process (70, 71). In the current study, PDC induced liver and renal injury through up-regulation of NF-κB/Il-6 and TLR4 expression, while, ARG or *L. plantarum* assisted with alleviating inflammation by reducing the liver and kidney NF-**κ**B, IL-1β, and TLR4 contents as compared with PDC rats. These results could be attributed to their anti-inflammatory mechanism; in addition, ARG in combination with *L. plantarum* exhibited synergistic effects as they returned the liver and kidney contents of NF-**κ**B, IL-1β, and TLR4 to their normal contents. In another study, ARG had an anti-inflammatory mechanism through the suppression of TNF-α and IL-6 in hepatic ischemia-reperfusion injury (72), also, probiotic *Lactobacillus* *casei Zhang* has an anti-inflammatory effect decreasing NF-κB/ TNF-α /TLR4 against liver injury induced by endotoxin- and d-galactosamine (73). Moreover, *L. plantarum* and ARG have an effect against liver injury provoked by LPS-induced liver injury in a mice model through decreasing TNF-α levels (74).

ROS have been linked to apoptosis as potential modulators, and oxidative stress has been linked to apoptosis. The ability of oxidative stress to induce apoptosis as a result of massive cellular damage has been linked to lipid peroxidation as well as changes in protein and nuclei (75, 76). Bax, a proapoptotic molecule, and Bcl-2, an antiapoptotic molecule, are both involved in cell death and are found in abundance in the mitochondria, nuclear membrane, and endoplasmic reticulum. Rupture and swelling of mitochondria, in response to apoptosis, may produce a large number of apoptosis-promoting factors. A Bcl-2 expression was down-regulated in acute liver and kidney injury (77). Immunohistochemical examination of hepatic and renal tissues of rats receiving PDC induced-acute hepatic and kidney injury showed elevated levels of Bax and Bcl-2 with a significant percentage increase of positively stained cells accompanied by strong and diffuse cytoplasmic staining of hepatocytes and renal tubules as compared with the normal control group. Conversely, combining ARG with *L. plantarum* revealed a significant decrease in Bax and an increase in Bcl-2 expression in the liver and kidneys of rats that received PDC induced-acute hepatic and kidney injury. 

To elucidate the role of ARG, *L. plantarum* and their combination in suppressing the apoptosis associated with PDC intoxication, this study exhibited the combining ARG with *L. plantarum* effect on hepatic and renal caspase-3 levels. Caspase-3 is a major apoptotic executor and a biomarker of cell death (78). PDC intoxication shifts the balance between pro- and anti-apoptotic signals toward the pro-apoptotic cascade, where, it affords up-regulation of Bax, and diminishes Bcl-2. It also induces the translocation of Bax from the cytosol to mitochondria releasing cytochrome c to the cytosol (79). Previous reports have regarded caspase-3 activation as a crucial cellular mechanism for the induction of apoptosis in hepatocytes and renal tubular cells in PDC-induced acute hepatic and renal injury (80). Combining ARG with *L. plantarum* significantly decreased the elevated hepatic and renal caspase-3 levels and restores the balance between pro- and anti-apoptotic signals. This implies that combining ARG with *L. plantarum* exerted their hepatic and neuroprotective effects through inhibition of ROS and TLR-4/ NF-κB -mediated apoptosis in rats intoxicated with PDC. Corroborating the current finding, prior studies documented that ARG in combination with *L. plantarum* exerted anti-apoptotic effects against endotoxin-induced liver injury in a rat model (81).

iNOS, on the other hand, is a pro-inflammatory enzyme that is highly expressed in the early stages of tissue inflammation (82). Recent studies have reported increased immunohistochemical iNOS expression following PDC administration, which is consistent with our findings (83). Following PDC administration, increased immunohistochemical iNOS expression was observed in hepatic and renal tissues, presumably due to oxidative stress and inflammation (84). Combining ARG with *L. plantarum* revealed individual sparse iNOS immune-positive cells due to the promising effect of *L. plantarum* to direct ARG, an NO precursor towards polyamine synthesis. It may be inferred that a meaningful synergy between *L. plantarum* and ARG may be expected in systemic impairments such as hepatic and liver dysfunctions.

Regarding histopathological investigations of hepatic and kidney, rats that received PDC in the present study have also revealed marked vaculized hepatocytes interspersed with abundant necrotic and apoptotic cells and extensive necrosis in renal tubules, and those findings were inconsistent with a previous study (85). Remarkable amelioration in the histopathological changes of hepatocytes and renal tubules in PDC rats pre-treated with ARG was evidenced by mild swelling and vacuolization of hepatocytes with normal renal tubules (86). Similarly, hepatocytes of PDC rats pre-treated with *L. plantarum* showed mild vacuolation of hepatocellular cytoplasm, with the presence of mitotic figure denoting regenerative activity in renal tubules. Combining ARG with *L. plantarum* could improve the histopathological picture of the liver and kidney of PDC rats, where hepatocytes and renal tubules appeared normal with normal round vesicular nuclei with normal pathological scores.

Daily oral administration of the dose of ARG (100 mg/kg), *Lactobacillus plantarum* (10^9^ CFU/ml), and their combination for 14 days, followed by induction of acute hepatic and kidney injury by single SC injection with PDC (20 mg/kg; day15). Forty eight hr later (day 17), blood samples were collected. Finally, rats were sacrificed for liver and kidney harvesting

**Table 1:** Pre-treatment effect of combining ARG with *Lactobacillus plantarum* on serum biochemical indices in rats receiving PDC induced-acute hepatic and kidney injury

Groups	AST ( IU/L )	ALT (IU/L )	ALP (IU/L)	**Serum Urea** **(mg/dl)**	Serum creatinine (mg/dl)	Serum Uric acid (mg/dl)
Normal	78.12±0.96	41.16±0.71	37.62±0.76	24.35±0.58	0.49±0.02	5.13±0.61
PDC	195.7±1.54^*^	94.97±1.59^*^	150.2±1.81^*^	76.83±1.04^*^	1.55±0.03^*^	8.75±0.09^*^
PDC+ARG	174.8±0.97^*@^	63.96±0.79^*@^	76.44±1.10^*@^	50.61±0.72^*@^	0.81±0.28^*@^	7.44±0.15^*@^
PDC+* Lactobacillus plantarum*	114.9±0.93^*@^	48.71±0.66^*@^	65.46±1.24^*@^	44.32±0.94^*@^	0.72±0.02^*@^	6.55±0.22^*@^
PDC+ARG+* Lactobacillus plantarum*	83.29±1.68^@^	43.29±1.14^@^	40.43±0.71^@^	26.78±0.86^@^	0.51±0.02^@^	5.29±0.14^@^
CDI	0.18	0.63	0.75	0.5	0.8	0.74

Acute hepatic and kidney injury was induced by single SC injection with PDC. Twenty four hr after the last dose of ARG (100 mg/kg), *Lactobacillus plantarum* (10^9^ CFU/ml) and their combination and serum biochemical indices were evaluated. Results are expressed as mean±SEM (n=8)

* Significant difference from normal control group *P<*0.05. ^@^ Significant difference from PDC received group. ^#^ represents significant synergism in CDI<1

**Table 2:** Pre-treatment effect of combining ARG with Lactobacillus plantarum on hepatic and renal oxidative stress biomarkers in rats receiving PDC induced-acute hepatic and kidney injury

**Parameter** **groups**	**GSH (mg/g.tissue)**		**MDA (nmol/mg)**		**Catalase (U/g. tissue)**	
	**Hepatic**	**Renal**	**Hepatic**	**Renal**	**Hepatic**	**Renal**
**Normal**	39.02±0.15	9.03±0.15	27.82±0.78	8.43± 0.42	572.9±2.385	470.9±2.721
**PDC**	14.19±0.31^*^	4.19±0.31^*^	58.39±0.69^*^	18.39±0.69^*^	417.9±3.97^*^	313.7±3.004^*^
**PDC+ARG**	35.26±0.41^*@^	7.66±0.21^*@^	33.84±0.29^*@^	10.84±0.29^*@^	553.2±1.701^*@^	475.3±1.198^*@^
**PDC+** *** Lactobacillus plantarum***	28.24±0.74^*@^	6.64±0.13^*@^	38.44±0.45^*@^	13.84±0.28^*@^	550.9±1.65^*@^	463.2±2.170^*@^
**PDC+** ***ARG+ Lactobacillus plantarum***	37.83±0.36^@^	8.43±0.18^@^	28.43±0.22^@^	8.83±0.32^@^	575.3±1.52^@^	449.9±1.515^@^
**CDI**	0.34	0.7	0.3	0.10	0.6	0.48

Acute hepatic and kidney injury was induced by single SC injection with PDC. Twenty four hr after the last dose of ARG (100 mg/kg), *Lactobacillus plantarum* (10^9^ CFU/ml) and their combination, hepatic and renal values of GSH, MDA, and catalase were evaluated. Results are expressed as mean±SEM (n=8)

*Significant difference from normal control group *P<*0.05. ^@^ Significant difference from PDC received group.^ #^ represents significant synergism in CDI<0.1

Acute hepatic and kidney injury was induced by a single SC injection with PDC. Forty eight hr after the dose of PDC, hepatic and renal levels of NF-κB were estimated. Results are expressed as mean±SEM (n=8). *Significant difference from normal control group *P<*0.05. ^@^ Significant difference from PDC received group. ^#^ Indicates synergistic interaction using coefficient drug index (CDI)

Acute hepatic and kidney injury was induced by a single SC injection with PDC. Forty eight hr after the dose of PDC, hepatic and renal levels of TLR- 4 were estimated. Results are expressed as mean±SEM (n=8). *Significant difference from normal control group *P<*0.05. ^@^ Significant difference from PDC received group. ^#^ Indicates synergistic interaction using coefficient drug index (CDI)

Acute hepatic and kidney injury was induced by a single SC injection with PDC. Forty eight hr after the dose of PDC, hepatic and renal levels of il-1β were estimated. Results are expressed as mean±SEM (n=8). * Significant difference from normal control group *P<*0.05. ^@^ Significant difference from PDC received group. ^#^ Indicates synergistic interaction using coefficient drug index (CDI)

Acute hepatic and kidney injury was induced by a single SC injection with PDC. Forty eight hr after the dose of PDC, hepatic and renal levels of TNF-α were estimated. Results are expressed as mean±SEM (n=8). * Significant difference from normal control group *P<*0.05. ^@^ Significant difference from PDC received group. ^#^ Indicates synergistic interaction using coefficient drug index (CDI)

**Table 3:** Pre-treatment effect of combining ARG with *Lactobacillus plantarum* on the total pathologic score recorded in the liver and kidneys of rats receiving PDC induced-acute hepatic and kidney injury

Renal pathologic score (mean±SE)	Hepatic pathologic score (mean±SE)	Groups
**0.10** ^c^ **±0.10**	0.20^c^±0.13	**Normal**
**3.00** ^a^ **±0.00**	2.60^a^±0.16	**PDC**
**0.40** ^b,c^ **±0.16**	0.80^b,c^±0.29	**PDC+ARG**
**0.60** ^b^ **±0.22**	0.90^b^±0.31	**PDC+** *** Lactobacillus plantarum***
**0.30** ^c^ **±0.15**	0.30^c^±0.15	**PDC+ARG+** *** Lactobacillus plantarum***
**0.3**	0.25	**CDI**

Acute hepatic and kidney injury was induced by single SC injection with PDC. Twenty four hr after the last dose of ARG (100 mg/kg), *Lactobacillus plantarum* (10^9^ CFU/ml) and their combination, hepatic and renal pathologic scores were recorded. Results are expressed as mean±SEM (n=8)

* Significant difference from normal control group *P<*0.05. ^@^ Significant difference from PDC received group. ^#^ represents significant synergism in CDI<1

Acute hepatic and kidney injury was induced by a single SC injection with PDC. Forty eight hr after the dose of PDC, hepatic and renal levels of Caspase-3 were estimated. Results are expressed as mean±SEM (n=8). *Significant difference from normal control group *P<*0.05. ^@^ Significant difference from PDC received group. ^#^ Indicates synergistic interaction using coefficient drug index (CDI)

Photomicrograph of liver tissue, stained with H&E, of (a, b) normal rats showing normal hepatocytes (a) with normal round vesicular nuclei (arrows)(b), (c, d) PDC group showing pronounced swelling of hepatocytes (c) with cytoplasmic reticulation and nuclear pyknosis (arrows), (d) in addition to abundant necrotic and /or apoptotic cells (red arrows-insert), (e, f) PDC+ARG group showing a pronounced decrease of hepatocellular swelling, (e) with mild vacuolar degeneration of hepatocytes (arrows)(f), (g, h) PDC+*Lactobacillus plantarum* group showing no evidence of cellular ballooning, (g) with mild vacuolation of hepatocellular cytoplasm (arrows) (h), (i, j) PDC+ARG+*Lactobacillus plantarum* group showing normal liver structure (i) with normal round vesicular nuclei (arrows) (j) (Stain: H&E; scale bar=100 µm)

**Table 4:** Pre-treatment effect of combining ARG with *Lactobacillus plantarum* on the expression of Bax, Bcl2, and iNOS in the liver and kidneys of rats receiving PDC induced-acute hepatic and kidney injury

iNOS expression kidney (% of positive cells/HPF)	iNOS expression liver (% of positive cells/HPF)	Bcl2 expression kidney (% of positive cells/HPF)	Bcl2 expression liver (% of positive cells/HPF)	Bax expression kidney (% of positive cells/HPF)	Bax expression liver (% of positive cells/HPF)	Groups
**0.00** ^d^ **±0.00**	0.00^d^±0.00	0.00^c^±0.48	1.00^c^±0.63	0.00^d^±0.00	0.00^d^±0.00	**Normal **
**4.00** ^a^ **±0.48**	4.00^a^±0.48	0.00^c^±0.31	0.00^d^±0.51	4.00^a^±0.51	4.00^a^±0.42	**PDC**
**2.00** ^b^ **±0.51**	2.00^b^±0.48	2.00^b^±0.73	2.00^a,b^±0.82	2.00^b^±0.67	2.00^b^±0.73	**PDC+ARG**
**2.00** ^b^ **±0.48**	2.00^b^±0.78	2.00^b^±0.73	2.00^b^±0.73	2.00^b^±0.63	2.00^b^±0.78	**PDC+** *** Lactobacillus plantarum***
**1.00** ^c^ **±0.66**	1.00^c^±0.48	3.00^a^±0.63	3.00^a^±0.94	1.00^c^±0.73	1.00^c^±0.51	**PDC+ARG+** *** Lactobacillus plantarum***
**0.2**	0.2	0.2	0.2	0.2	0.2	**CDI**

Acute hepatic and kidney injury was induced by single SC injection with PDC. Twenty four hr after the last dose of ARG (100 mg/kg), *Lactobacillus plantarum* (10^9^ CFU/ml) and their combination, the expression of Bax, Bcl2, and iNOS in the liver and kidneys of rats were recorded. Results are expressed as Median±SD (n=8). *Significant difference from normal control group *P<*0.05. ^@^ Significant difference from PDC received group. ^#^ Represents significant synergism in CDI<1

photomicrograph of renal tissue, stained with H&E, of (a, b) normal rats showing normal renal glomeruli (G) and tubules (a) with normal epithelial lining (arrows) (b), (c, d) PDC group showing extensive renal damage with extensive necrosis of renal tubules (c) with small deep bluish pyknotic nuclei (arrows), in addition to tubular dilatation (D)(d), (e, f) PDC+ARG group showing normal renal tubules (e) with no evidence of tubular necrosis (arrow) (f), (g, h) PDC+*Lactobacillus plantarum* group showing normal renal tubules (g) with the presence of mitotic figure (arrow)(h), (i, j) PDC+ARG+*Lactobacillus plantarum* group showing normal renal structure (i) with normal renal tubules (arrow)(j) (Stain: H&E; scale bar=100µm)

photomicrograph of immunohistochemically-stained liver tissue of (a,b,c) normal rats showing no Bax expression (a), few Bcl-2 positively stained cells (b) and no iNOS expression (c), (d, e, f) PDC group showing increased percentage of Bax-positively stained cells with strong brown cytoplasmic and/or nuclear staining (d), sparse Bcl-2 weakly stained cells (e) and numerous intense iNOS immune-positive cells (f), (g, h, i) PDC+ARG group showing significant reduction of percentage of Bax-positively stained cells (g), significant increase of percentage of Bcl-2 positively stained cells (h) and significant decrease of percentage of iNOS-positively stained cells (i), (j, k, l) PDC+*Lactobacillus plantarum* group showing few Bax-positively stained cells (j), increased Bcl-2 positively stained cells (k) and few iNOS-positively stained cells (l), and (m, n, o) PDC+ARG+*Lactobacillus plantarum* group showing scant Bax weakly stained cells (m), numerous strong Bcl-2-positively stained cells (n) and individual sparse iNOS immune-positive cells. (Bax, Bcl-2, and iNOS immunohistochemical staining; scale bar=100µm)

Photomicrograph of immunohistochemically-stained renal tissue, of (a, b, c) normal rats showing no Bax expression (a), no Bcl-2 positively stained cells (b) and no iNOS expression (c), (d, e, f) PDC group showing extensive Bax-positively stained cells with strong brown cytoplasmic staining (d), sparse Bcl-2 weakly stained cells in the renal glomeruli (e) and numerous intense iNOS immune-positive cells (f), (g, h, i) PDC+ARG group showing significant reduction of percentage of Bax-positively stained cells (g), significant increase of percentage of Bcl-2 positively stained cells (h) and significant decrease of percentage of iNOS-positively stained cells (i), (j, k, l) PDC+*Lactobacillus plantarum*-treated rats showing weakly stained Bax-immune positive cells (j), increased Bcl-2 positively stained cells in the renal glomeruli and tubules (k) and few iNOS-positively stained cells (l), and (m, n, o) PDC+ARG+*Lactobacillus plantarum *group rats showing scant Bax weakly stained cells (m), numerous strong Bcl-2-positively stained cells (n) and individual sparse iNOS immune-positive cells. (Bax, Bcl-2, and iNOS immunohistochemical staining; scale bar=100 µm)

## Conclusion

The present study indicates that ARG and/or *L. plantarum* mitigate acute liver and kidney injury in rats exerting their beneficial effects through their antioxidant and anti-inflammatory properties via suppressing iNOS and TLR 4/ NF-κB signaling pathway and antiapoptotic ability through caspase-3 and Bax/Bcl2. This alternative bacteriotherapy combination may be considered a potential safe protective agent against hepatic and renal injury caused by PDC and are innovative and cost-effective.

## Authors’ Contributions

AAS participated in practical experimentation, carried out the statistical analyses, and wrote the manuscript. AS participated in the practical experimentation and wrote and revised the final manuscript. AH carried out all the histopathological and immunohistochemical studies.

## Ethics approval and consent to paticipate

The animal experiments were performed according to the recommendations in the Guide for the Care and Use of Laboratory Animals of the National Institutes of Health (NIH No. 85.23: revised 1985) and approved by the Medical Research Ethics Committee (MREC) of the National Research Centre (Egypt), under approval number (5413042022).

## Conflicts of Interest

The authors declare no conflicts of interest exist.
